# Structural and Functional Aspects of Ebola Virus Proteins

**DOI:** 10.3390/pathogens10101330

**Published:** 2021-10-15

**Authors:** Sahil Jain, Ekaterina Martynova, Albert Rizvanov, Svetlana Khaiboullina, Manoj Baranwal

**Affiliations:** 1 Department of Biotechnology, Thapar Institute of Engineering and Technology, Patiala 147004, Punjab, India; drsahiljain88@gmail.com; 2 University Institute of Biotechnology, Chandigarh University, Mohali 140413, Punjab, India; 3 Institute of Fundamental Medicine and Biology, Kazan Federal University, 420008 Kazan, Tatarstan, Russia; katerinamarty@yandex.ru (E.M.); albert.rizvanov@kpfu.ru (A.R.); sv.khaiboullina@gmail.com (S.K.)

**Keywords:** Ebola virus, Ebola virus proteins, Ebola virus protein functions

## Abstract

Ebola virus (EBOV), member of genus *Ebolavirus*, family *Filoviridae*, have a non-segmented, single-stranded RNA that contains seven genes: (a) nucleoprotein (NP), (b) viral protein 35 (VP35), (c) VP40, (d) glycoprotein (GP), (e) VP30, (f) VP24, and (g) RNA polymerase (L). All genes encode for one protein each except GP, producing three pre-proteins due to the transcriptional editing. These pre-proteins are translated into four products, namely: (a) soluble secreted glycoprotein (sGP), (b) Δ-peptide, (c) full-length transmembrane spike glycoprotein (GP), and (d) soluble small secreted glycoprotein (ssGP). Further, shed GP is released from infected cells due to cleavage of GP by tumor necrosis factor α-converting enzyme (TACE). This review presents a detailed discussion on various functional aspects of all EBOV proteins and their residues. An introduction to ebolaviruses and their life cycle is also provided for clarity of the available analysis. We believe that this review will help understand the roles played by different EBOV proteins in the pathogenesis of the disease. It will help in targeting significant protein residues for therapeutic and multi-protein/peptide vaccine development.

## 1. Introduction

**Figure 1:**
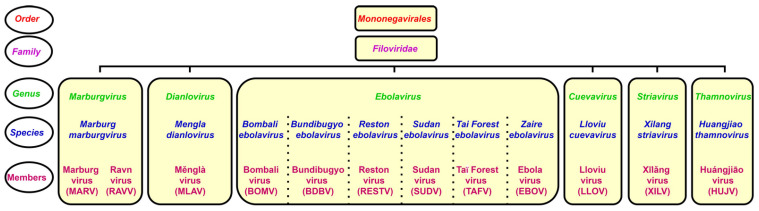
Taxonomical classification of ebolaviruses.

The genus *Ebolavirus* belongs to the family *Filoviridae* and consists of six identified species. Schematic taxonomy classification of *Filoviridae* according to the International Committee on Taxonomy of Viruses (ICTV) is presented in Figure 1 [1,2]. Recently, a new genus, *Dianlovirus*, has been proposed by Yang and co-workers, including the virus circulating in Chinese bats, named Měnglà virus (MLAV) (Figure 1) [3,4]. According to the current terminology, the Ebola virus (EBOV) is a *Zaire ebolavirus* species in the *Ebolavirus* genus. Most of the members of the genus *Ebolavirus*, except Reston virus (RESTV) and Bombali virus (BOMV), cause severe and frequently fatal hemorrhagic fever in humans and non-human primates (NHPs). In contrast, BOMV infects bats exclusively while RESTV is known to be pathogenic for humanized mice, though no human infection has been detected as yet [1,2,5].

EBOV has a thread-like shape virion, which can be changed to circular or filamentous [6]. The filamentous shape could appear as long, short, branched, unbranched, or forming “6” and “U” configurations [7]. The viral genome is 19 kb long, linear, non-segmented negative sense (NNS), single-stranded RNA, encoding seven genes [8,9] (Table 1). Each gene, except GP, contains a single open reading frame (ORF). In contrast, the GP gene consists of three overlapping ORFs [10,11]. During the assembly, viral RNA forms a ribonucleoprotein (RNP) complex with NP, L, VP30, VP35 and VP24 [12], which appears as a helical nucleocapsid (NC) [13,14]. NC protects the viral RNA from degradation by endonucleases and hosts immune response [14,15].

In the last 45 years, ebolavirus outbreaks with varying fatalities have been documented mainly in Africa, resulting in over 15,200 deaths [16]. The rising frequency of episodes has led to better disease management measures and vaccine development efforts worldwide [17,18,19,20,21]. On 19 December 2019, the Ervebo vaccine, based on recombinant vesicular stomatitis virus (VSV) expressing EBOV GP, received approval from the Food and Drug Administration (FDA) as the first licensed vaccine against EBOV [22,23]. However, multiple obstacles, such as high frequency side effects, difficulties to manufacture, high cost, low immunogenicity, and lack of a global outreach, interfere with efficacy of EBOV outbreak control [24,25]. This review will provide a comprehensive analysis of all EBOV proteins functions and enlist the protein residues involved. This review will identify therapeutic and multi-protein/peptide vaccine development targets by understanding viral proteins’ role in the replication cycle.

## 2. Ebola Virus Life Cycle

**Figure 2:**
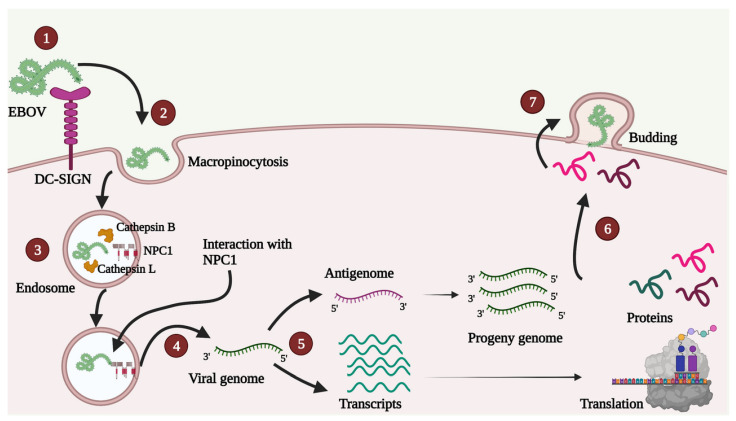
A simple diagrammatic representation of various steps in the EBOV life cycle. 1. Attachment—EBOV can interact with different host cell receptors, and none of the receptors is indispensable for attachment. In the Figure, DC-SIGN receptor is shown as an example. 2. Uptake—Uptake mainly occurs by micropinocytosis, as shown, though other methods such as clathrin-mediated endocytosis and caveolin-mediated endocytosis are also contemplated. 3. Entry—GP1 proteolysis inside endosome enables viral interaction with obligate host receptor cholesterol transporter Niemann-Pick C1 (NPC1; shown in red color). 4. Release—After membrane fusion, the viral genome is released in the host cell cytoplasm. 5. Transcription and Replication—Primary transcription occurs in the host cell cytoplasm followed by a translation. Antigenome is used as a template for synthesis of progeny genomes. 6. Transport—Various proteins are transported near the plasma membrane. 7. Assembly and Budding—VP40 plays a crucial role in assembly, virus-like particle (VLP) formation and budding.

A schematic depiction of the viral life cycle is presented in Figure 2.

### 2.1. Attachment and Entry

To enter the host cell, EBOV can use several attachment factors, such as human folate receptor- α [38], β1 integrins [39], TYRO3 receptor tyrosine kinase family members [40], T-cell immunoglobulin, and mucin domain 1 (TIM1) [41]. Additionally, various lectins, such as dendritic cell-specific intercellular adhesion molecule-3-grabbing non-integrin (DC-SIGN), liver/lymph node-specific ICAM-3 grabbing non-integrin (L- SIGN), and human macrophage galactose and N-acetylgalactosamine-specific C-type lectin (hMGL) [42,43,44] were shown to serve as entry receptors. None of these receptors are indispensable for EBOV attachment [45,46,47], which could include explainability of the virus to target various cell types.

Upon binding to the receptor, EBOV enters the host cells via three mechanisms: (a) Macropinocytosis [48], (b) Clathrin-mediated endocytosis [49,50], and (c) caveolin-mediated endocytosis [51]. The internalization mechanism appears related to the shape of the virus. Currently, macropinocytosis is believed to be the primary uptake mechanism [52,53,54], while a combination of different mechanisms is also suggested [55,56,57,58].

EBOV GP consists of two subunits, GP1 and GP2 (discussed later). After uptake, proteolysis of EBOV GP1 appears significant for viral entry [47]. The mechanism of proteolysis varies depending on the host cell type [59] and can be carried out by cathepsin B, cathepsin L [60,61], as well as thermolysin [62]. This EBOV GP1 proteolysis is essential for viral interaction with the obligate host receptor cholesterol transporter Niemann-Pick C1 (NPC1), a step critical for viral entry [63,64]. This interaction initiates the fusion of viral and host cell membrane, leading to the release of viral RNP into the cytoplasm (Figure 2) [46].

### 2.2. Transcription and Replication

NP encapsidates both, filoviral genome, as well as anti-genome [65]. NP associated RNA acts as a template for viral RNA transcription and replication [66]. Primary transcription and translation take place in the host cell cytoplasm (Figure 2). Accumulation of NP and other EBOV proteins results in the formation of inclusion bodies, which serve as additional sites for transcription and replication [67,68,69]. Replication is initiated at a promoter region of viral RNA that flanks the transcription initiation sequence of the first EBOV gene [70]. Many host factors, such as DNA topoisomerase I (TOP1) [71], RNA-binding protein Staufen 1 [72], and RNA splicing and export factors Nuclear RNA export factor 1 (NXF1) and TEDx-box helicase 39B (DDX39B) [73], are essential for transcription and replication. Importantly, transcription and replication is regulated by the phosphorylated state of VP30 (discussed later).

**Figure 3:**
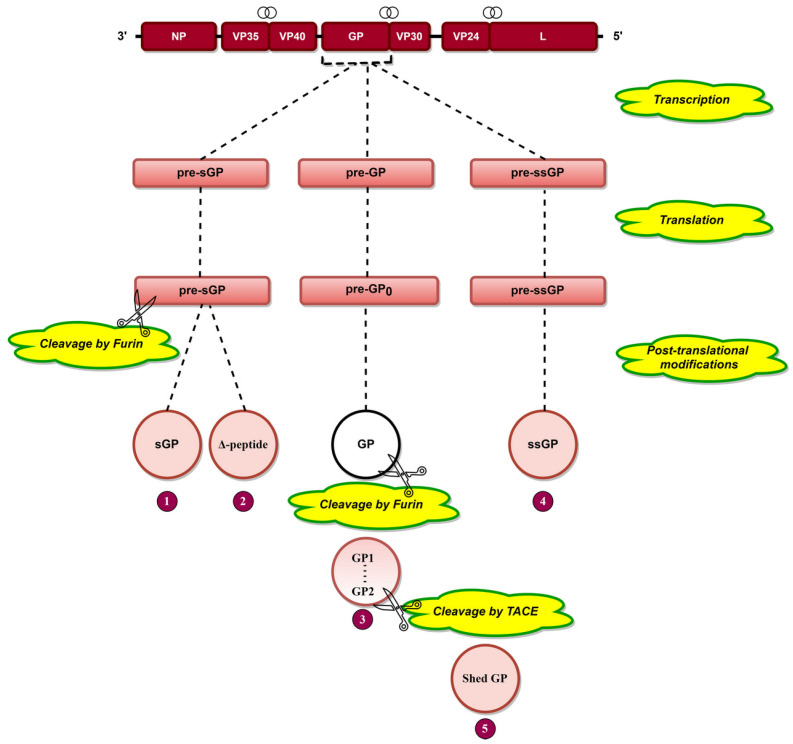
The arrangement of EBOV genes is presented at the top, where the symbol 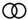 indicates the overlapping genes. Transcriptional editing of the GP gene by L protein results in three RNA transcripts: pre-sGP, pre-GP, and pre-ssGP. Translation of these three transcripts results in three pre-proteins, namely, pre-sGP, pre-GP0, and pre-ssGP, respectively. Pre-sGP is cleaved post-translationally by furin into sGP and Δ-peptide. pre-GP_0_ and pre-ssGP result in full-length transmembrane spike glycoprotein (GP) and soluble small-secreted glycoprotein (ssGP). GP is cleaved by furin into GP1 and GP2 subunits held together by disulfide bonds. Shed GP is released from infected cells due to cleavage of GP by tumor necrosis factor α-converting enzyme (TACE).

Transcriptional editing of the GP gene [29] results in the generation of three transcripts, pre-sGP, pre-GP, and pre-ssGP (Figure 3), which are translated into pre-sGP pre-GP_0_ and pre-ssGP, respectively [74]. Pre-sGP is cleaved post-translationally by furin into sGP and Δ-peptide [30]. Pre-GP_0_ forms GP post-translationally, cleaving furin into GP1 and GP2 subunits linked by disulfide bonds (Figure 3) [31,75,76]. Shed GP is released from infected cells due to cleavage of GP at aa 637 by tumor necrosis factor α-converting enzyme (TACE) (Figure 3) [77]. GP is heavily glycosylated (discussed later), a phenomenon significant for EBOV pathogenesis.

### 2.3. Assembly and Budding

At the late stage of transcription, the RNP complex, GP, and VP40 are transported to the cell surface via different mechanisms. Transport of RNP employs actin [78,79], while GP is carried to the cell surface via secretory pathway, where it is glycosylated [80], as well as cleaved into GP1 and GP2 subunits [76]. VP40 trafficking requires interaction with IQ motif containing GTPase activating protein 1(IQGAP1) [81], coat protein complex II (COPII) [82], microtubules [83,84], or actin [85,86].

## 3. Ebola Virus Proteins and Their Functions

### 3.1. Nucleoprotein (NP)

**Figure 4:**
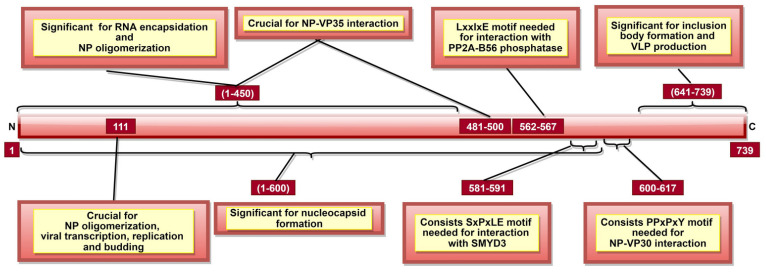
A schematic representation of EBOV nucleoprotein (NP). NP aa 1–450 are involved in RNA encapsidation, NP oligomerization, nucleocapsid (NC) as well as NP-VP35 interaction (along with aa 481–500). aa 562–567 and 581–591 consist of LxxIxE and SxPxLE motifs, respectively, interacting with different host proteins. PPxPxY motif amongst aa 600–617 is significant for NP-VP30 interaction. Interaction of these three motifs with their respective targets is significant to regulate viral transcription.

EBOV NP is a multifunctional protein, contributing to NC and RNP formation [12,13,14]. It was shown that aa 1–600 (Figure 4) are crucial for NC formation and viral replication [87]. Additionally, aa 1–450 are involved in RNA encapsidation/ssRNA binding [12,13,88] and NP oligomerization [13,87,89] (Table 2). NP oligomerization facilitates NP-ssRNA interaction, which is essential for NC formation [89,90]. A recent study indicated the significance of aa 111 in NP oligomerization, viral transcription, and replication [91]. Another study highlighted the significance of NP C-terminal domain (CTD) aa 641–739 (Figure 4) information of inclusion bodies and infectious virus-like particle (VLP) production [92]. Interestingly, laboratory data indicate that only point mutations in NP and L are required for virus adaptation to different species [93].

Additionally, various post-translational modifications of NP are documented. It was shown that NP undergoes O-glycosylation and sialylation, which are significant for NC formation [87,94] as they facilitate direct NP-VP35 and NP-VP24 interaction [94]. NP-VP35 interaction involves aa 1–450 of NP (Table 2) [88,90]. This interaction regulates viral RNA synthesis by chaperoning NP in a monomer state, preventing its binding to ssRNA [88,90]. Recently, NP central domain aa 481–500 (Figure 4) were also suggested to be essential for NP-VP35 interaction [92]. Additionally, NP aa 2–150 and 601–739 were shown to be involved in NP-VP40 interaction, which is significant for the recruitment of NP into VLP [95]. Additionally, the PPxPxY motif, especially aa residues 600–617 (Figure 4), is responsible for NP-VP30 interaction, which is essential for viral RNA transcription [96,97].

Recent studies indicate that NP could interact with host cell proteins to facilitate virus transcription and replication. It was shown that NP recruits host factor carbamoyl-phosphate synthetase 2, aspartate transcarbamylase, and dihydroorotase in an RNA-independent manner to facilitate EBOV genome transcription and replication [98]. Additionally, LxxIxE motif of NP (aa 562–567) interacts with host PP2A-B56 phosphatase, which dephosphorylates NP-bound-VP30 and enables viral transcription [99]. Another NP motif, SxPxLE (aa 581–591) (Figure 4), recruits host SET and MYND domain-containing protein 3 (SMYD3), which regulates viral transcription by increasing the NP-VP30 interaction in a dose-dependent manner [100]. Additionally, Wendt et al. proposed that NP could recruit host nuclear RNA export factor 1 (NXF1), a component of nuclear mRNA export pathway, to facilitate viral mRNA transport from inclusion bodies [101]. Another host protein, HSP70, was reported to maintain NP stability, enabling viral replication, as NP degraded in its absence [102]. NP also interacts with RUVBL (RuvB-like) 1 and RUVBL2 proteins in an RNA-independent manner forming the R2TP complex required for capsid assembly [103].

The critical NP residues discussed above are summarized in Table 2. It could be suggested that NP is essential for viral replication and transcription. Additionally, NP is vital for RNA encapsidation, NC formation and capsid assembly. NP, in part, completes these functions via interaction with EBOV (except GP) and host proteins. Therefore, a therapeutic or peptide/protein vaccine candidate targeting critical NP aa and motifs (Table 2, Figure 4) could hinder NC formation and NP-host protein interactions, abrogating virus transcription and virus assembly.

### 3.2. Viral Protein 35 (VP35)

Tetrameric VP35 is functionally analogous to other NNS RNA viruses [8,27,134]. VP35 is crucial for viral transcription and replication [134] and possesses NTPase and helicase activities, suggesting that it could affect transcription via NTP hydrolysis and NTP-dependent unwinding of RNA helices, respectively [135]. VP35 also contributes to genome packaging [136] and nucleocapsid assembly [13,14] as it binds the monomeric state of NP to prevent premature and non-specific encapsidation of viral RNA.

**Figure 5:**
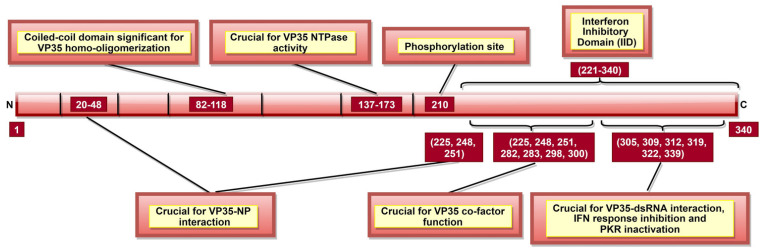
A schematic representation of EBOV viral protein 35 (VP35). VP35 aa 137–173 are significant for VP35 NTPase activity, which affects viral transcription and rep-lication. Interferon inhibitory domain (IID; aa 221–340) consists of aa 225, 248, and 251 which are involved in two roles: VP35-NP interaction (along with N-terminal aa 20–48) and VP35 co-factor function (along with IID aa 282, 283, 298, and 300). Phosphorylation of aa 210 regulates VP35-NP interaction, as well as viral transcription and replication.

VP35 is crucial for host immune response evasion, where host anti-viral defense is inhibited in multiple ways. It can suppress host interferon (IFN) response in both dsRNA-binding-dependent and dsRNA-binding-independent manners. In this respect, the VP35 CTD region, in particular aa 221–340, were shown to function as IFN inhibitory domain (IID) [109] or RNA-binding domain (RBD) [110] (Figure 5). It was demonstrated that specific residues within IID (Table 2, Figure 5) are required to bind VP35 to viral dsRNA [107,110]. This interaction is crucial for the IFN inhibiting function of VP35, as it blocks viral dsRNA recognition by retinoic-acid-inducible gene I (RIG-I) and melanoma differentiation-associated protein 5 (MDA-5), the intracellular pattern recognition receptors [109].

It was shown that VP35 could also inhibit IFN regulatory factor 3 (IRF-3) dimerization, phosphorylation and nuclear localization [137]. VP35 can do it by impairing the ability of TANK-binding kinase 1 (TBK-1) and IκB kinase epsilon (IKKε) kinases to interact with IRF-3 [138]. VP35 can also suppress IFN transcription by increasing SUMOylation of IRF-7 via interaction with protein inhibitors of activated STATs 1 (PIAS1) [139]. IID of VP35, especially aa 239, 312, and 322, also blocks the protein activator of IFN-induced protein kinase (PACT), thus, preventing activation of PACT-induced RIG-I ATPase [106]. Further, VP35 aa 304–340 (Figure 5) can inactivate protein kinase R (PKR; an antiviral protein), which enables continuous viral protein synthesis [108].

Interaction of VP35 with other viral proteins is significant for multiple purposes. VP35-L interaction requires VP35 homo-oligomerization, completed by N-terminal aa 82–118 (Figure 5) [105]. VP35 could also function as a non-enzymatic co-factor for the L protein [107], where several IID constituent residues are critical (Table 2, Figure 5) [104]. It appears that the co-factor function is independent of IID binding to dsRNA [107], but the latter can still modulate VP35-NP interaction [104]. VP35 residues 20–48, 225, 248, and 251 (Table 2) are significant for VP35-NP complex formation and regulate viral RNA synthesis [88,104]. A recent study reported the role of VP35 phosphorylation, especially at aa 210 (Figure 5), in the regulation of VP35-NP interaction, as well as viral transcription and replication [140].

To conclude, VP35 plays a chief role in host immune evasion by blocking viral dsRNA recognition by host immune receptors, inhibiting IRF-3 and TBK-1/ IKKε complex formation, increasing IRF-7 SUMOylation, preventing RIG-I ATPase activation and inactivating PKR (Table 2, Figure 5). Moreover, VP35 is suggested to play a role in NC formation, viral transcription, replication, and genome packaging. Therefore, targeting VP35 might enable the host to mount a more robust immune response and disturb the viral structural integrity.

### 3.3. VP40

**Figure 6:**
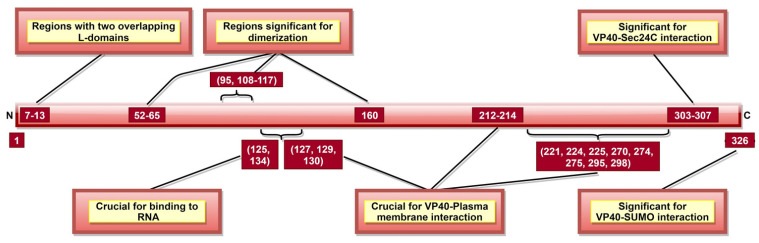
A schematic representation of EBOV viral protein 40 (VP40). VP40 aa 7–13 consists of two late budding domains (L-domains), namely PTAP (aa 7–10) and PPEY (aa 10–13). Both L-domains are crucial to viral budding. VP40 aa 52–65, 95, 108–117, and 160 are involved in dimerization, while aa 303–307 enables VP40–Sec24C interaction. Both the dimerization state and the interaction with Sec24C are significant for proper cellular trafficking of VP40. The binding of octameric VP40 to ssRNA (via VP40 aa 125 and 134) negatively regulates transcription. aa 326 provides stability to VP40 via interaction with host SUMO protein.

VP40, the most abundantly expressed protein [28], is essential for viral assembly and budding [141]. VP40 aa 292–295 (Table 2) were reported as critical for VLP production and controlled inhibition of viral transcription, as a mutation in 292–295 aa sequence altered these functions [116]. A recent study suggested that aa 326 (Figure 6) is involved in SUMO–VP40 interaction which is significant for VP40 stability [142]. Additionally, VP40 contains two late budding domains (L-domains) located at aa 7–10 (PTAP) and aa 10–13 (PPEY) (Figure 6) [143], interacting with host proteins. PTAP makes a complex with tumor susceptibility gene 101 protein (tsg101) [144], while PPEY binds to ubiquitin ligase, neuronal precursor cell-expressed developmentally downregulated 4 (Nedd4) [145], as well as to ITCH E3 ubiquitin ligase [146]. The PTAPtsg101 interaction helps recruit VP40 into lipid raft domains on the plasma membrane [143], while the PPEY–Nedd4 complex covalently ubiquitinates viral matrix proteins, which is required for virus budding [145]. These data strongly suggest that L-domains are essential for budding [147] but limited in viral replication [148].

VP40 is classified as a transformer protein [149,150], as it could obtain multiple conformational states: dimers, hexamers, filaments and octamers [151]. VP40 dimerizes in solution with the help of aa 95 and 160 [111], as well as NTD aa 52–65 and 108–117 [112] (Table 2, Figure 6). Dimerized confirmation is essential for proper trafficking of VP40, where aa 303–307 (Figure 6) form VP40-Sec24C complex facilitating intracellular transport to the plasma membrane [82]. Dimers can also assemble into filaments via CTD interactions between two dimers, where aa 241 and 307 (Table 2) are shown to be critical. This state is essential for proper matrix assembly and budding [112].

NTD aa 127, 129 and 130 [114] and aa 212–214 [115] (Figure 6) are required for proper VP40 localization to the plasma membrane, oligomerization and budding. The interaction with the plasma membrane is mediated by various VP40 CTD residues (Table 2, Figure 6) [112]. Additionally, aa 213, 295, and 298 facilitate deep penetration of VP40 into the plasma membrane [152]. The VP40 dimers at the plasma membrane are then oligomerized into linear VP40 hexamers [112,153]. Both deep penetration and hexamer formation are critical to viral assembly and budding [112,152]. A recent study highlighted the significance of aa 191 for effective VP40 localization to the plasma membrane, oligomerization and VLP formation [154].

In addition to dimers, filaments, and hexamers, VP40 can form an octameric ring configuration where NTD plays the leading role [112]. This configuration and aa 125 and 134 (Figure 6) were essential for VP40-ssRNA binding [112,113]. The latter is implicated in the negative regulation of transcription [111].

These data indicate that the main function of VP40 is in viral assembly and budding (Table 2, Figure 6). Therefore, targeting critical VP40 aa might help suppress viral spread after infection by hindering the budding process. Additionally, it is suggested to play a role in viral transcription inhibition and interaction with the host cell plasma membrane.

### 3.4. Glycoprotein

#### 3.4.1. GP

**Figure 7:**
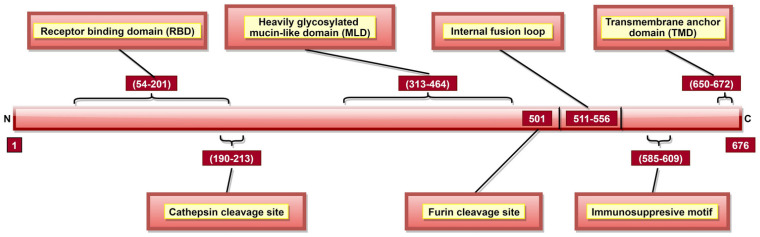
A schematic representation of EBOV glycoprotein (GP). GP aa 54–201 form the receptor-binding domain (RBD) or receptor binding site (RBS) responsible for attachment to host cell-surface receptors. Cathepsin cleavage site is present in aa 190–213, and proteolysis via cathepsins is significant for viral infectivity. GP is cleaved at aa 501 by furin into GP1 and GP2 subunits. The immunosuppressive motif (aa 585–609) plays a role in bystander lymphocyte apoptosis and cytokine dysregulation. The transmembrane anchor domain (TMD; aa 650–672) helps tether GP onto the viral surface.

GP is a type I transmembrane fusogenic protein, which is translated only 20% of time by the GP gene as it can be cytotoxic to certain cell types [29,31]. As the only EBOV protein present on the viral surface, GP is responsible for pathogenic differences of ebolaviruses [123]. In 2010, it was reported that GP could cause “steric occlusion”, a phenomenon which interferes with immune recognition of HLA class I and II molecules [155]. In other studies, it was shown that aa 159, 160, 162, 170 [120], and 214–270 [119] (Table 2) provide protein stability, while aa 55, 57, 63, and 64 are involved in membrane fusion-mediated conformational changes [123]. GP has a cathepsin cleavage site at aa 190–213 (Table 2, Figure 7) [123,124], which is proteolyzed inside the endosome, a step critical for viral infection [60,61]. GP is post-translationally cleaved by furin at aa residue 501 (Figure 7), resulting in GP1 and GP2 subunits linked by disulfide bonds [31,75,76]. These heterodimers form trimeric viral peplomer [31].

GP1 mediates attachment to host cells using the receptor-binding site (RBS) located at aa 54–201 (Figure 7) [122]. Studies have identified multiple aa (Table 2) as critical for viral entry [117,118,119,120,121,156]. Among these, aa 64, 95 [121] and 114, 115, 140 [124] are involved in direct contact with host cell receptors while aa 43, 54, 56, 60, 61 and 79 contribute to post-binding steps of viral entry [121]. A Mucin-like domain (MLD) was identified in aa 313–464 (Figure 7) [120], which does not directly impact viral entry [32] but can stimulate host dendritic cells by activation of mitogen-activated protein kinase (MAPK) and nuclear factor kappa B (NF-κB) pathways [157].

GP2 contributes to the fusion of viral and host cell membranes [158]. An internal fusion loop at aa 511–556 position (Figure 7) [120] consists of hydrophobic residues (Table 2) inserted into the target cell membrane [123]. GP2 also has a transmembrane anchor domain (TMD; aa 650–672) (Figure 7) [31] which helps to tether GP onto the viral surface [74]. Interestingly, GP2 also has the anti-tetherin activity, promoting VP40-mediated viral budding by the host cell and disabling the immune response stimulation via NF-κB signaling [159,160]. An immunosuppressive motif (aa 585–609; Figure 7) located near the C-terminal [29,31] can cause lymphocyte apoptosis, as well as cytokine dysregulation during EVD [126].

Post-translational N-linked glycosylation of GP results in a thick coating of oligosaccharides, protecting viral GP against host humoral immune response [123] and promoting protein expression and function [161]. In total, 15 N-linked and 80 O-linked glycosylation sites have been identified in GP1 [32,161]. GP2 has 2 N-linked glycosylation sites (aa 563 and 618; Table 2) [75] critical for GP processing, oligomerization and confirmation [125]. Once a threshold amount of viral GP is present on the host cell plasma membrane, the highly glycosylated MLD masks specific cell-surface proteins, such as major histocompatibility complex (MHC) class I, αVβ3, etc., in a cell-dependent manner [155,162,163,164]. MHC class I masking protects the virus from host CD8^+^ T cell recognition [155]. Additionally, MLD masks self GP epitopes, shielding GP from the host immune recognition [155,162].

#### 3.4.2. Soluble Secreted Glycoprotein (sGP)

sGP is the primary GP gene product, sharing 295 N-terminal aa with the full-length transmembrane spike glycoprotein (GP) [31]. Six N-glycosylation sites (aa 40, 204, 228, 238, 257, and 268) [165] and one C-mannosylation site (aa 288) [166] have been identified in sGP. It is a non-structural protein (NSP) [4], though a structural role was contemplated in a study, wherein sGP substituted for GP1, producing a functional sGP-GP2 protein [167]. A potential contribution of sGP in viral dissemination was suggested by Bradley et al., demonstrating inhibition of pro-inflammatory cytokines production from non-infected macrophages and impairing chemotaxis of activated macrophages [168]. sGP also has an anti-inflammatory capacity to restore the endothelial cell barrier function dysregulated by GP [165,169]. It was also shown that sGP contributes to host immune evasion by acting as a decoy for anti-GP antibodies [170]. In a recent study, detection of serum sGP was suggested as a biomarker for Ebola virus disease (EVD) diagnosis as large quantities of this protein are found in blood at the early stages of the disease [171].

#### 3.4.3. Δ-Peptide

Δ-peptide is a non-structural, secretory product of the GP gene, composed of 40 aa [30]. It is a O-glycosylated, sialylated peptide [30] rich in cationic and aromatic residues [172]. It can inhibit viral entry into filovirus-permissive cells, preventing superinfection [172,173]. In 2015, Gallaher et al. reported the role of Δ-peptide as a viroporin, based on presence of a lytic sequence motif [172,174]. In another study, Δ-peptide was shown to indiscriminately permeabilized mammalian cells in culture, supporting its role as a viroporin [175].

#### 3.4.4. Shed GP

Like sGP, shed GP is believed to function as “antibody sinks” [32] as they compete with GP for antibody binding [176]. It is also believed that shed GP helps to reduce the cellular cytotoxicity caused by GP [177]. Additionally, shed GP activates non-infected dendritic cells and macrophages [177], leading to massive cytokine production and increased vascular permeability [177].

#### 3.4.5. Soluble Small Secreted Glycoprotein (ssGP)

ssGP is an NSP composed of 298 aa [32] (Table 1), with 295 N-terminal aa identical to sGP and GP [10]. It is secreted as a N-glycosylated homodimer formed by intermolecular disulfide bond at aa 53 [10]. Function of ssGP still remains unknown [171].

To summarize, GP, as the only protein on the viral surface, is responsible for viral entry, i.e., attachment to host cells and fusion of viral and host cell membrane. GP–NPC1 interaction is an indispensable step for viral infection, and, therefore, targeting GP shall hinder the viral infection. Additionally, GP assists in viral budding, while GP and shed GP contribute to the cytokine storm. Further, GP, sGP, and shed GP enable host immune evasion, while sGP contributes to viral spread and may be used as a biomarker for early diagnosis of EVD. Therefore, targeting various GP gene products might be beneficial in the development of an effective vaccine.

### 3.5. VP30

**Figure 8:**
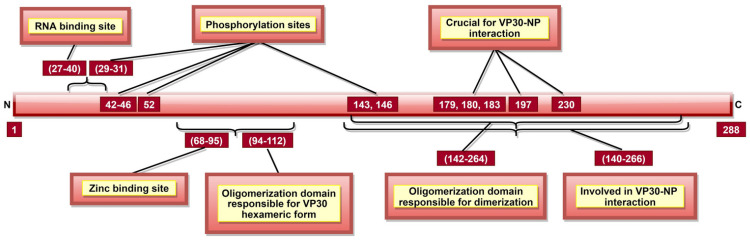
A schematic representation of EBOV viral protein 30 (VP30). aa 68–95 comprise of zinc-binding site. Zinc is important for VP30’s transcriptional functions and VP30–RNA interaction via VP30 RNA binding site (aa 27–40). aa 143 and 146 are involved in three roles: (a) phosphorylation sites, (b) dimerization (aa 142–264), and (c) VP30–NP interaction (aa 140–266).

VP30 is a structural, hexameric phosphoprotein composed of three dimers [33,129]. Dimers are formed by aa 142–264, while aa 94–112 (Figure 8) are required to produce hexameric form [129,178]. VP30 aa 27–40 (Figure 8) form a disordered, non-hydrophobic, arginine-rich region interacting with viral RNA [127]. VP30 is indispensable for RNA transcription initiation [128]. Interestingly, mutations at aa 179, 180, and 183 render VP30 incapable of transcription initiation, suggesting their importance in this process [129].

Factors impacting the role of VP30 in transcription initiation are RNA secondary structure formation, VP30–NP interaction, zinc-binding and VP30 phosphorylation. The NP gene transcription starts signal forms a stem-loop like secondary structure, essential for VP30-dependent transcription initiation [179]. The VP30 aa 140–266 (Table 2, Figure 8) are responsible for VP30–NP complex formation [96,97,129], and a threshold level of interaction is required for optimal viral transcription, below and beyond which transcriptional activity is flawed [96]. Further, VP30 role in regulating viral RNA transcription requires a zinc-binding site located at aa 68–95 (Figure 8) [128].

VP30 phosphorylation mainly occurs at serine (aa 29–31, 42, 44, and 46) and threonine (aa 52, 143, and 146) residues (Figure 8) [33,180]. Low or un-phosphorylated VP30 seems responsible for transcription initiation of all seven genes in the EBOV genome [181,182]. Whether low or un-phosphorylated VP30 is required as a transcription factor depends on the virus replication stage. Additionally, if low phosphorylated, a constant phosphorylation/dephosphorylation within the same VP30 molecule is required for effective transcription initiation [183]. Amongst the phosphorylation sites, aa 29 seems to be the most critical as it can solely execute all VP30 transcription functions [183]. Complete VP30 phosphorylation at all serine residues between aa 29–46 abrogates transcription initiation function [184].

The impact of phosphorylation on VP30-NP interaction was extensively debated over the past two decades [33,181,182]. The latest consensus states that phosphorylation leads to a more robust interaction with NP which allows VP30 to be associated with NC and enter newly synthesized virus particles, wherein the un-phosphorylated VP30 can initiate transcription [182,183]. Overall, it is accepted that the phosphorylation state of VP30 is dynamic and modulated by the virus to achieve an intricate balance between transcription and replication processes, which happen simultaneously during the EBOV life cycle [181,184].

The above discussion suggests that VP30 chiefly plays a role in viral transcription initiation via its zinc-binding, NP interaction and phosphorylation characteristics (Table 2, Figure 8). Abrogating this function shall hinder the production of multiple RNA copies. Therefore, VP30 is a plausible candidate for therapeutic and vaccine development studies targeting primary transcription in the host cell cytoplasm.

### 3.6. VP24

**Figure 9:**
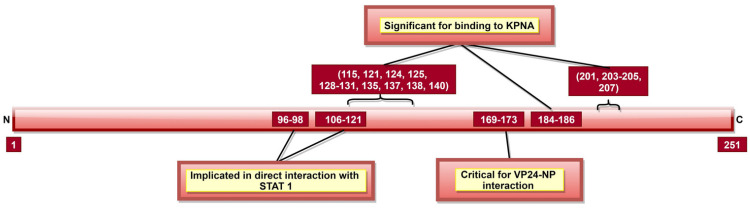
A schematic representation of EBOV viral protein 24 (VP24). VP24 aa 169–173 facilitate interaction with NP, which is crucial for nucleocapsid (NC) formation, EBOV replication cycle and egress phase. Tyrosine-phosphorylated STAT1, hnRNP C1/C2 and VP24 interact with KPNA at the same position; therefore, upon VP24–KPNA interaction, transport of tyrosine-phosphorylated STAT1 to the nucleus is stopped as well as translocation of hnRNP C1/C2 from the cytoplasm to the nucleus is partially prevented.

VP24 makes up approximately 7.5% of total EBOV proteins and is one of the five proteins involved in EBOV NC formation. VP24 aa 169–173 (Table 2, Figure 9) interact with NP, which is indispensable for NC formation and completion of the EBOV replication cycle [133]. Additionally, VP24, together with VP35 and NP, is responsible for packaging NC into virions as it induces favorable conformational changes in the NC. This structural change also signals the end of replication and the beginning of the egress phase [133,185].

Like VP35, VP24 inhibits host immune response using several mechanisms [186]. VP24 inhibits IFN responses by blocking p38 phosphorylation and hampering the p38 MAPK pathway [187]. It can also block activation of NF-κB, which has multiple IFN responsive genes targeted downstream [188]. IFN inducible pathways could also be inhibited by arresting the nuclear translocation of tyrosine-phosphorylated STAT1 via VP24 interaction with NPI-1 subfamily members of karyopherin-α (KPNA, also called importin-α) [132]. Additionally, the role of aa 96–98 and 106–121 (Figure 9) indirect interaction of VP24 with un-phosphorylated STAT1 was reported [130,131]. VP24 aa 142–147 and 26–50 (Table 2) were reported as necessary to facilitate binding to KPNA1 [130,132]. Multiple other aa (Table 2, Figure 9) were also shown responsible for VP24–KPNA5 interaction [131]. Further, it was reported that oxidative stress effects the VP24 modulation of the host response, facilitating recovery of infected cells from stress [189].

It appears that the central role of VP24 is in host immune evasion, carried out by inhibiting p38 MAPK and NF-κB pathway activation. VP24 also seems critical to viral replication and is involved in budding viral initiation. Therefore, targeting these residues (Table 2) during vaccine development may hinder viral replication.

### 3.7. L Protein

**Figure 10:**
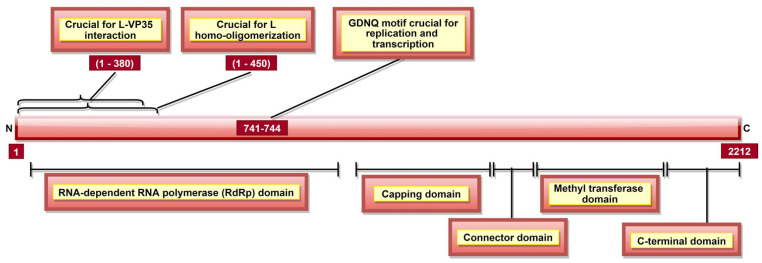
A schematic representation of EBOV L protein. It has five domains, namely, (a) RdRp domain responsible for polyadenylation and transcription/replication (via GDNQ motif), (b) capping domain with PRNTase activity which caps mRNA co-transcriptionally via transfer of a GDP molecule, (c) connector domain, (d) methyltransferase domain involved in methylation of capped mRNA, as well as cap-independent methylation of internal mRNA adenosine, and (e) C-terminal domain responsible for the regulation of methylation activities of methyltransferase domain. L aa 1–380 are involved in L-VP35 interaction, enabling L’s re-localization into viral inclusion bodies.

L, a part of the RNP complex [36], is the largest, multi-subunit and multifunctional EBOV protein composed of 2212 aa [36] (Table 1). There are five domains in L protein (Figure 10), namely, (a) RNA-dependent RNA polymerase (RdRp) domain with transcription/replication and polyadenylation activity, (b) capping domain with polyribonucleotidyl transferase (PRNTase) activity, (c) connector domain (CD) with an organizational role, (d) a methyltransferase domain with MTase activity, and e) a small C-terminal domain (Table 3, Figure 10) [190]. In addition, EBOV L residues 1–450 contain a homo-oligomerization domain (Figure 10) [105]. L protein aa 1–380 (Figure 10) are involved in L-VP35 interaction, which happens in a non-competitive manner and does not require L homo-oligomerization [105]. The L-VP35 binding enables re-localization of L into viral inclusion bodies. In addition to VP35, The RESTV L protein was shown to interact with VP30 [191].

L protein is essential for virus replication, yet little is known about its other functions, mainly due to its large size and lack of specific antibodies [192,193]. The current understanding of this protein’s function comes from studies using vesicular stomatitis virus (VSV), as L protein is highly conserved and homologous amongst *Mononegavirales* members [65,190,194,195]. Therefore, the L domains functions presented here are based on reports made using VSV L and not always the EBOV.

A GDNQ motif has been reported as a catalytic center in several negative sense RNA polymerases. In 2017, The EBOV RdRp domain was also reported to consist of the GDNQ motif within aa residues 741–744 (Figure 10), which is crucial for viral replication and transcription [193]. The RdRp domain is involved in the polyadenylation of L mRNA [196]. The capping of viral transcripts occurs co-transcriptionally via the transfer of a GDP molecule by PRNTase to 5′ phosphate of mRNA [197]. Then, the first nucleotide and guanosine of the capped mRNA become methylated at 2′-O and N-7 positions, respectively, by MTase activity [198]. Additionally, a cap-independent, internal adenosine 2′-O methylation activity of methyltransferase domain in SUDV L protein (aa 1693–2036) was demonstrated [194,199]. The small CTD of SUDV L protein located at aa 2037–2210 plays a critical role in RNA recruitment for methylation [194]. It also regulates cap methylation (aa 2043, 2067, 2068, 2118, 2189, and 2193) and internal methylation (aa 2043, 2067, 2068, 2112, 2113, 2118, 2189, and 2193) activities of methyltransferase domain [194].

## 4. Conclusions and Future Perspectives

It is imperative to understand proteins functions with respect to the EBOV life cycle and pathogenesis to identify practical and specific protein therapeutic targets. This review summarizes data on aa residues of EBOV proteins involved in essential functions, such as viral entry, host immune evasion, replication, transcription, and budding. A cross-reactive, multi-protein/peptide vaccine candidate developed by utilizing the information presented in this review could help curb various characteristic EVD symptoms and affect different stages of the EVD life cycle.

Multiple proteins and aa sites were highlighted in our review, which have a high potential for vaccine development. For instance, NC formation and viral replication could be hindered by collectively targeting NP aa 110–400 and VP24 aa 169–173. Replication and its regulation could also be affected by targeting VP35 aa 210 and L aa 741–744. Viral transcription could be affected by targeting NP aa 562–567, NP aa 600–617, and VP30 aa 140–266, while transcription regulation can be blocked by collectively targeting NP aa 240–383, VP35 aa 20–48, and VP30 aa 29–183. Moreover, targeting NP aa stretch 550–600 might disable multiple NP-host-protein interactions significant for transcription and regulation. Viral ingress may be blocked by targeting GP aa 54–213 as it affects GP stability, interaction with obligate host receptor (NPC1) and membrane-fusion mediated conformational changes. A critical step for viral spread is host immune evasion, accomplished with the help of VP35, sGP, shed GP and VP24 proteins. This could be prevented by targeting specific regions of these proteins, such as VP30 aa 221–340 and VP24 aa 120–190. Moreover, as aa 1–295 are identical in GP, sGP, and ssGP; therefore, therapeutic or vaccine candidates against GP aa 54–213 could prove effective against sGP (primary GP gene product), as well as ssGP. This will be effective in preventing viral entry, as well as in curbing host immune evasion. Three proteins contribute to viral egress: VP40, GP, and VP24. GP2 displays anti-tetherin activity, while VP24 causes favorable structural changes in NC to signal viral egress initiation. As VP40 is primarily involved in assembly and budding, targeting aa 212–275, a region significant for localization to and interaction with the plasma membrane (Table 2), may hinder the budding process.

Multiple studies have analyzed EBOV proteins viz., NP [20,200,201,202], VP35 [203,204], VP40 [203], GP [21,205,206,207,208,209,210,211,212,213], and VP24 [214,215] potential as therapeutics and vaccine candidates. In December 2019, Ervebo, a recombinant vesicular stomatitis virus (VSV) vector-based vaccine, was approved by FDA as the first vaccine against the Ebola virus [216]. Currently, nearly 14 potent Ebola vaccine candidates are in various phases of clinical trials [217]. Still, the presence of non-immunogenic, unwanted components in traditional vaccines, allergic, toxic, or autoimmune reactions, the requirement for BSL-4 facility and probability of a reversal of attenuated virus state [218,219,220] impede EBOV vaccine development efforts. Clearly, GP has been the major focus for vaccine development, while less focus was placed on other viral proteins. Therefore, further studies will facilitate our understanding of the EBOV proteins potentials to protect from infection. Key motifs of these proteins might be analyzed to find potential epitopes. These identified epitopes might be estimated for the generation of T and B cell immune response and their binding affinity with human leukocyte antigen (HLA). Further, immunodominant epitopes belonging to different EBOV proteins may be linked to form one multi-peptide vaccine construct. The immunogenic potential of the construct might be validated in different animal models, before considering it for clinical trials. Targeting the key domains of EBOV proteins outlined in this review or their combination could be an approach for development of future EBOV vaccine which is globally effective.

## Figures and Tables

**Table 1:** Ebola virus genes and their products.

Genes	Pre-Proteins	Proteins	Length of Protein (aa)	Weight of Protein (kDa)	Reference
Nucleoprotein (NP)		NP	739	83.31	[26]
* VP35		VP35	340	37.37	[8,27]
VP40		VP40	326	40	[28]
Glycoprotein (GP)	pre-sGP	Soluble secreted glycoprotein (sGP)	364	50	[29,30]
		Δ-peptide	40	10–14	[30]
	pre-GP_0_	Full-length transmembrane spike glycoprotein (GP)	676	150	[29,31]
	pre-ssGP	Soluble small secreted glycoprotein (ssGP)	298	30	[10,32]
VP30		VP30	288	30	[33]
VP24		VP24	251	24	[34,35]
RNA polymerase (L)		L	2212	253	[36,37]

* VP: Viral protein.

**Table 2:** A summary of various critical functions performed by various EBOV proteins and their amino acids involved.

Protein	Amino Acids (aa)	Function	Reference
NP	1–450, especially 160, 171, 174, 298, 310 and 401	RNA encapsidation/ssRNA binding	[12,13,88]
	1–450, especially 110, 349, 373, 374, 382 and 383	NP oligomerization; significant for nucleocapsid (NC) formation	[13,87,89]
	1–450 (especially 244–383, critically 240, 248 and 252) and 481–500	NP-VP35 interaction; significant for viral RNA synthesis regulation	[88,90]
	2–150 and 601–739	NP-VP40 interaction; significant for recruiting NP into VLP	[95]
	562–567	NP-PP2A-B56 phosphatase interaction; significant for enabling viral transcription	[99]
	581–591	Recruiting host SET and MYND domain-containing protein 3 (SMYD3); significant for viral transcription regulation	[100]
	600–617	NP-VP30 interaction; significant for viral RNA transcription	[96,97]
	641–739	Inclusion body and virus-like particle (VLP) formation	[92]
VP35	20–48, 225, 248 and 251	VP35-NP interaction; significant for viral RNA synthesis regulation	[88,104]
	82–118	VP35 homo-oligomerization; significant for VP35-L interaction	[105]
	221–340, especially 239, 312 and 322	Interaction with protein activator of IFN-induced protein kinase (PACT); significant to prevent activation of PACT-induced RIG-I ATPase	[106]
	225, 248, 251, 282, 283, 298 and 300	Enable VP35 to function as a non-enzymatic co-factor for the L protein	[104,107]
	304–340	Inactivating protein kinase R (PKR); significant for continuous viral protein synthesis	[108]
	305, 309, 312, 319, 322 and 339	Binding to dsRNA; significant to protect dsRNA from recognition by host immune receptors	[107,109,110]
VP40	52–65, 95, 108–117 and 160	VP40 dimerization; significant for VP40 cellular trafficking	[111,112]
	125 and 134	Octameric VP40 and ssRNA binding; significant for negative transcription regulation	[112,113]
	127, 129, 130 and 212–214	Significant for VP40 localization to the plasma membrane, oligomerization and budding	[114,115]
	221, 224, 225, 270, 274 and 275	VP40 interaction with plasma membrane	[112]
	241 and 307	VP40 filaments formation; significant for assembly and budding	[112]
	292–295	Significant for VLP production and controlled viral transcription inhibition	[116]
	303–307	VP40-Sec24C interaction; significant for internal trafficking of VP40 to plasma membrane	[82]
GP	43, 52, 54, 56, 57, 60, 61, 63, 64, 66, 79, 82, 88, 95, 114, 115, 140, 143, 146, 147, 153, 154, 159, 170 and 181	Significant for viral entry	[117,118,119,120,121]
	54–201	Receptor-binding site	[122]
	55, 57, 63 and 64	Involved in membrane fusion-mediated conformational changes	[123]
	159, 160, 162, 170 and 214–270	GP stability	[119,120]
	190–213, especially aa 190, 193 and 194	Cathepsin cleavage site; significant for viral interaction with the obligate host receptor	[123,124]
	529, 531, 533, 534, 535 and 537	Hydrophobic residues which insert into the target cell membrane	[123]
	563 and 618	2 N-linked glycosylation sites; significant for GP processing, oligomerization and functioning	[75,125]
	585–609	Immunosuppressive motif; cause lymphocyte apoptosis and cytokine dysregulation.	[29,31,126]
VP30	27–40	VP30-ssRNA interaction	[127]
	68–95	Zinc-binding site; significant for transcription regulation	[128]
	140–266	VP30-NP interaction; significant for viral transcription	[96,97,129]
	179, 180 and 183	Significant for transcription initiation	[129]
VP24	96–98 and 106–121	VP24-unphosphorylated STAT1 interaction	[130,131]
	115, 121, 124, 125, 128–131, 135, 137, 138, 140, 184–186, 201, 203–205 and 207	VP24-KPNA5 interaction	[131]
	142–147 and 26–50, especially 36–45	VP24-KPNA1 interaction	[132]
	169–173, critically 170 and 171	VP24-NP interaction; significant for NC formation and viral replication	[133]

**Table 3:** Various domains of L protein and their functions.

Domain	Function
RNA-dependent RNA polymerase (RdRp) domain	Transcription/replication and polyadenylation activity
Capping domain	Polyribonucleotidyl transferase (PRNTase) activity
Connector domain (CD)	Organizational role
Methyltransferase domain	MTase activity
C-terminal domain	RNA methylation regulation

## Data Availability

The datasets generated during and/or analysed during the current study are available from the corresponding author on reasonable request.
